# Nanophase-separated Ni_3_Nb as an automobile exhaust catalyst†
†Electronic supplementary information (ESI) available: Demonstration procedure, experimental and characterization details. See DOI: 10.1039/c6sc05473k



**DOI:** 10.1039/c6sc05473k

**Published:** 2017-03-13

**Authors:** Toyokazu Tanabe, Tsubasa Imai, Tomoharu Tokunaga, Shigeo Arai, Yuta Yamamoto, Shigenori Ueda, Gubbala V. Ramesh, Satoshi Nagao, Hirohito Hirata, Shin-ichi Matsumoto, Takeshi Fujita, Hideki Abe

**Affiliations:** a Department of Material & Life Chemistry , Kanagawa University , Yokohama 221-8686 , Japan; b National Institute for Materials Science , 1-1 Namiki , Tsukuba , Ibaraki 305-0044 , Japan . Email: abe.hideki@nims.go.jp; c Graduate School of Science and Technology , Saitama University , 255 Shimo-Okubo , Saitama 338-8570 , Japan; d Ecotopia Science Institute , Nagoya University , Nagoya 464-8603 , Japan; e Synchrotron X-ray Station at SPring-8 , National Institute for Materials Science , 1-1-1 Kouto , Sayo , Hyogo 679-5148 , Japan; f Toyota Motor Corporation , Mishuku 1200 , Susono , Shizuoka 410-1107 , Japan; g WPI Advanced Institute for Materials Research , Tohoku University , Sendai 980-8577 , Japan . Email: tfujita@wpi-aimr.tohoku.ac.jp

## Abstract

Catalytic remediation of automobile exhaust has relied on precious metals (PMs) including platinum (Pt). Herein, we report that an intermetallic phase of Ni and niobium (Nb) (*i.e.*, Ni_3_Nb) exhibits a significantly higher activity than that of Pt for the remediation of the most toxic gas in exhaust (*i.e.*, nitrogen monoxide (NO)) in the presence of carbon monoxide (CO). When subjected to the exhaust-remediation atmosphere, Ni_3_Nb spontaneously evolves into a catalytically active nanophase-separated structure consisting of filamentous Ni networks (thickness < 10 nm) that are incorporated in a niobium oxide matrix (*i.e.*, NbO_*x*_ (*x* < 5/2)). The exposure of the filamentous Ni promotes NO dissociation, CO oxidation and N_2_ generation, and the NbO_*x*_ matrix absorbs excessive nitrogen adatoms to retain the active Ni^0^ sites at the metal/oxide interface. Furthermore, the NbO_*x*_ matrix immobilizes the filamentous Ni at elevated temperatures to produce long-term and stable catalytic performance over hundreds of hours.

## Abstract 2

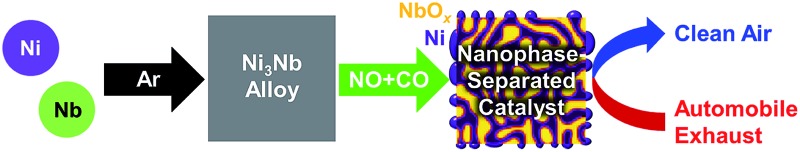
Nanophase-separated Ni_3_Nb alloy exhibited higher performance than traditional Pt catalysts toward the remediation of automobile exhaust.

## Introduction

The environmental impact of automobile exhaust is becoming a challenge not only for rising countries but also for the global community.^1–3^ Remediation of automobile exhaust relies on catalytic converters consisting of precious metals (PMs) including platinum (Pt). The consumption of PMs is increasing to meet the demands of ground transportation in emerging countries and the tighter emissions limits in advanced countries.^4^ Moreover, PM catalysts are required by different technologies, such as polymer-electrolyte membrane fuel cells.^5^ The development of PM-free catalytic materials is highly desirable to reduce the use of PMs in the automobile industry due to possible depletion of PMs in the near future.^6^

Earth-abundant 3d metals including nickel (Ni) were once considered for use as automobile exhaust catalysts.^7,8^ However, none of these 3d metals were realistic alternatives to PMs primarily due to their inherent susceptibility to dissociative adsorption of nitrogen oxides (NO_*x*_) in the exhaust.^9,10^ Indeed, the adsorption energies of oxygen and nitrogen atoms to the Ni surface are, 470 kJ mol^–1^ (O_ad_–Ni(111))^9^ and 470 kJ mol^–1^ (N_ad_–Ni(111)),^10^ respectively, which is 10–15% larger than the corresponding values for the Pt surface (*i.e.*, 405 kJ mol^–1^ (O_ad_–Pt(111))^9^ and 430 kJ mol^–1^ (N_ad_–Pt(111))), respectively.^10^ The 3d metals are readily deactivated when subjected to the exhaust atmosphere because the adatoms form stable blocking layers over the surface that inhibit further catalysis (*i.e.*, catalyst poisoning).^11^ Another drawback of the 3d metals for exhaust remediation is their low stability to thermal cycles. Automobile exhaust catalysts are assembled into catalytic converters in the form of supported nanoparticles (particle size < 10 nm).^12,13^ The 3d metal nanoparticles are prone to particle agglomeration *via* the Ostwald ripening process because their melting points are close to the operation temperatures of catalytic converters (<300 °C for four-stroke gasoline engines).^14,15^

Herein, we report that a nanophase-separated structure emerging from an intermetallic phase of Ni and niobium (Nb) (*i.e.*, nanophase-separated Ni_3_Nb) exhibits significantly higher catalytic performance than that of Pt catalysts for the remediation of one of the most toxic chemical species in exhaust (*i.e.*, nitrogen monoxide (NO)) in the presence of carbon monoxide (CO).^16^ Microscopic investigations including *in situ* transmission electron microscopy have demonstrated that the catalytic performance of nanophase-separated Ni_3_Nb is ultimately attributed to filamentous Ni and a NbO_*x*_ matrix, which spontaneously emerges on the intermetallic surface in the reaction atmosphere due to selective oxidation of Nb. The NbO_*x*_ matrix absorbs nitrogen adatoms from the Ni surface to retain the active Ni^0^ sites for improved NO remediation activity. Moreover, the filamentous Ni forms an agglomeration-tolerant network inside and/or over the surface of the NbO_*x*_ matrix, which results in long-term stable NO remediation for more than 500 hours.

## Results and discussion

A Ni_3_Nb intermetallic precursor (average particle size: 50 μm) was obtained by powdering Ni_3_Nb ingots synthesized by arc-melting elemental metals in a pure argon atmosphere (see Fig. S1 and S2† for characterization data of the different intermetallics including Ni_3_Nb: powder X-ray diffraction (pXRD)^17^ and hard X-ray photoemission spectra (HAXPES)). The powder of the elemental metals, such as Ni, Pt, Nb and Nb_2_O_5_, was used as purchased as the control catalysts. Nb_2_O_5_-supported Ni nanoparticles were also synthesized and used as a control (see ESI† for details).

**Fig. 1:**
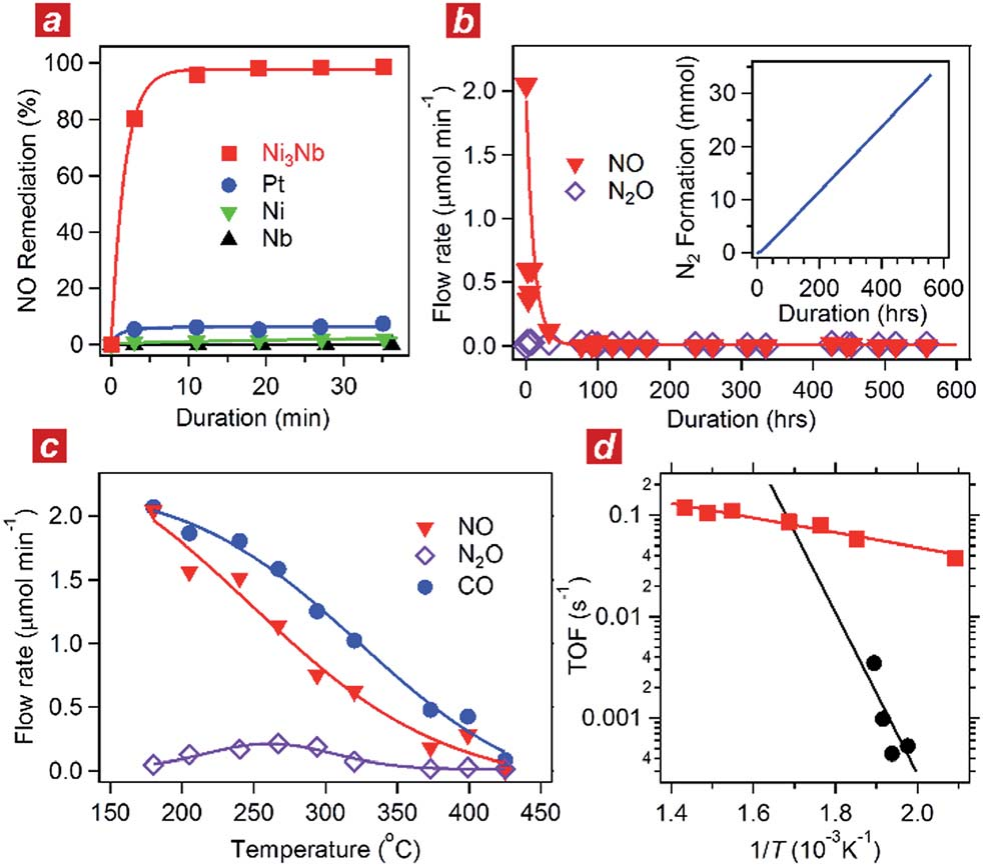
Catalytic performance of the Ni_3_Nb catalyst. (a) Time course of NO remediation at 325 °C over the Ni_3_Nb (red), Pt (blue), Ni (green) and Nb (black) catalysts. (b) Long-term NO remediation over the Ni_3_Nb catalyst at 400 °C in a steady flow of the He-balanced reactant gas. Inset shows the generation of N_2_ as a function of duration. (c) Concentrations of NO (red), N_2_O (purple) and CO (blue) in the effluent gas presented as functions of temperature. (d) Turnover frequencies (TOFs) of the Ni_3_Nb catalyst (red) and commercial Pt catalysts (black) for NO remediation plotted as functions of inverse temperature.

Each of the catalysts was transferred into a gas-circulation reactor (see Experimental section). An aliquot consisting of 10 kPa of the reactant gas (NO : CO = 1 : 1) was circulated through the catalyst powder at 325 °C. Fig. 1a shows the time course of the gas composition over the Ni_3_Nb, Pt, Ni and Nb materials. Pt exhibited a finite NO remediation activity, reaching 7.5% NO remediation 35 min after exposure to the reactant gas. Neither Ni nor Nb promoted NO remediation in 60 min. The Ni_3_Nb material exhibited a much higher NO remediation activity than that of Ni, Nb or even Pt, and a NO remediation of 96% was achieved in 11 min.

Fig. 1b shows the results of a long-duration catalytic test for the Ni_3_Nb material in a steady flow of a He-balanced reactant gas (5 ml min^–1^; NO : CO : He = 1 : 1 : 98; 400 °C; feeding rate = 5 cm^3^ min^–1^; space velocity = 30 000 h^–1^). Neither NO nor N_2_O was detected in the effluent gas 50 h after the exposure to the catalyst. The Ni_3_Nb material promoted NO remediation for more than 500 hours, and this catalyst converted 66 mmol of NO to N_2_ (inset of Fig. 1b). Fig. 1c shows the result of catalytic tests conducted at different temperatures after the long-duration test. The CO concentration monotonously decreased with increasing temperature and reached zero at 425 °C. The NO concentration decreased more steeply than the CO concentration because a portion of the NO was converted to nitrous oxide (N_2_O) in a temperature range from 175 to 375 °C *via* the following reaction path: NO + 1/2CO = 1/2N_2_O + 1/2CO_2_. The NO_*x*_ fraction in the effluent gas disappeared at 425 °C or higher temperatures, where the reaction proceeded *via* the major path (*i.e.*, NO + CO = 1/2N_2_ + CO_2_).

Fig. 1d shows the turnover frequency (TOF) of the Ni_3_Nb material for NO remediation as a function of the inverse of the temperature, which was calculated from the data in Fig. 1c. The number of active sites on the sample surface was determined to be 0.47 μmol g^–1^ by CO chemisorption at 298 K. The TOF of the Ni_3_Nb material was calculated to be 0.075 s^–1^ at 300 °C, which was higher than that for the commercial Pt catalysts (0.025 s^–1^) and even higher than that reported for the state-of-the-art Pt–Rh catalysts (0.047 s^–1^).^18–20^ The Ni_3_Nb material can be employed as a rational substitute for traditional PM catalysts for NO remediation based on the high TOF and long-term stability.

**Fig. 2:**
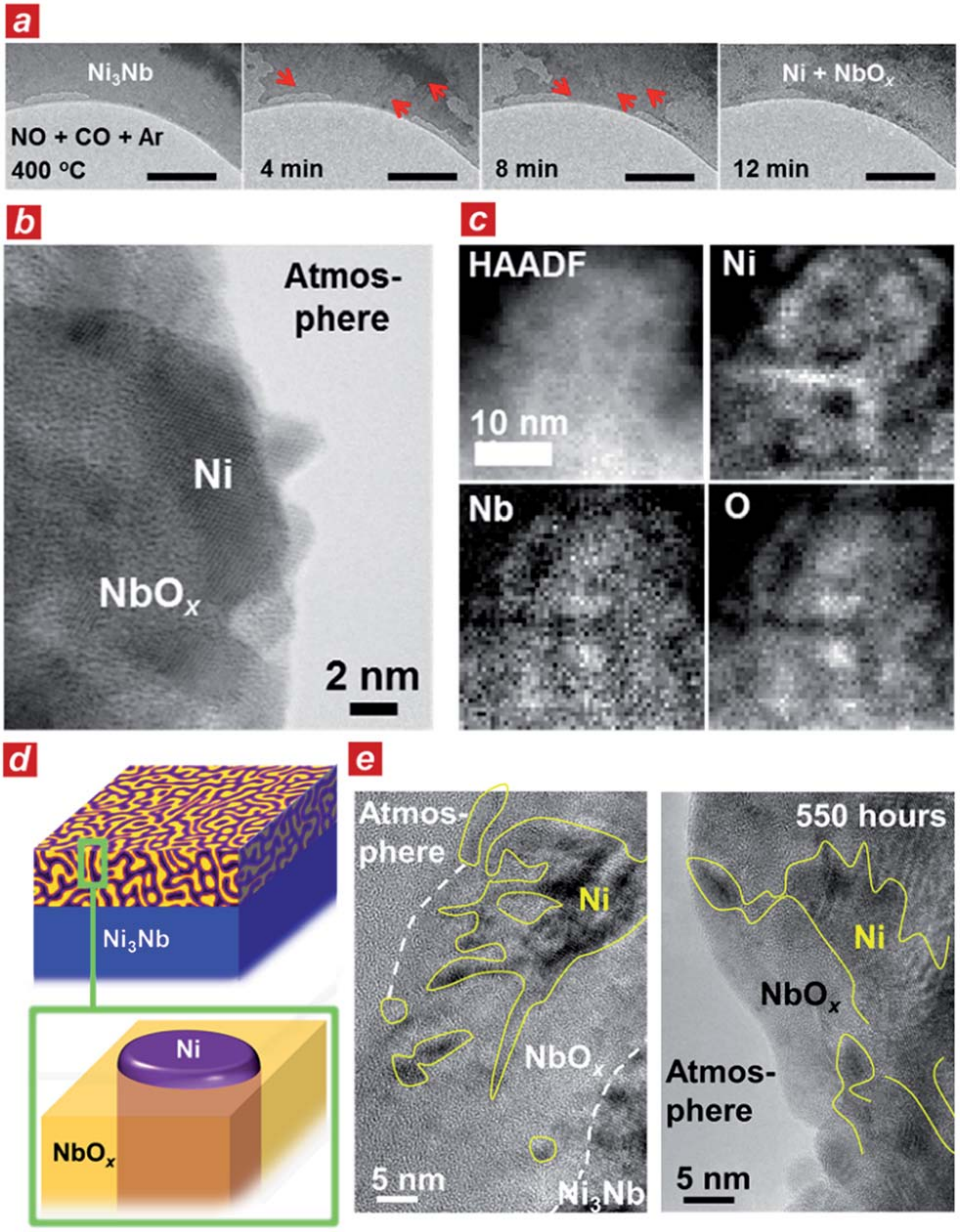
Microscopic characterizations. (a) Evolution of nanophase-separated structures on the Ni_3_Nb surface exposed to the Ar-balanced reactant gas at 400 °C. The scale bars correspond to 200 nm. (b) Scanning transmission microscope (STEM) image of the nanophase-separated Ni_3_Nb catalyst. (c) Annular-dark field (HAADF) image and elemental-mapping images of the nanophase-separated Ni_3_Nb catalyst. (d) Structural model of the nanophase-separated Ni_3_Nb catalyst. (e) Cross-sectional transmission electron microscope (TEM) images of the nanophase-separated Ni_3_Nb catalyst after 0.5 h (left) and 550 h of exposure to the reactant gas at 400 °C (right).

To elucidate the origin of the catalytic performance of the Ni_3_Nb material, we conducted microscopic investigations including *in situ* transmission electron microscopy.^21^ As shown in Fig. 2a, a low-contrast phase propagated from both the top-left and bottom-right corners of the Ni_3_Nb material after exposure to the reactant gas (NO : CO : Ar = 1 : 1 : 98) at 400 °C (red arrows show the propagation direction of the low-contrast phase; see ESI† for the *in situ* TEM observation). This low-contrast phase consisted of Ni metal and low-crystalline, oxygen-deficient NbO_*x*_ (*x* < 5/2) with a crystal structure that was identical to that of Nb_2_O_5_ (see Fig. S3 and S4† for HAXPES and Fig. S5† for pXRD^22^).

The Ni_3_Nb material after the catalysis was further characterized using high-resolution transmission electron microscopes (TEM and STEM). As expected from the HAXPES and pXRD analyses, the surface layer of the material was consisted of Ni metal and low-crystalline NbO_*x*_ after the gas exposure. Importantly, the Ni phase was not dispersed as isolated nanoparticles on the NbO_*x*_ surface (Fig. 2b, see also Fig. S6†). As shown in the elemental-mapping images using electron energy loss spectroscopy (EELS; Fig. 2c), Ni formed a nanometer-thick filamentous network inside the bulk and/or over the surface to result in a nanophase-separated Ni_3_Nb, and the distribution of this network was exclusive to the distributions of Nb or O.

Based on the TEM characterizations, a structural model of the catalytic centre of the nanophase-separated Ni_3_Nb catalyst has been determined (Fig. 2d). When exposed to the reaction atmosphere, the intermetallic Ni_3_Nb evolves into a nanophase-separated structure that consists of filamentous Ni and a NbO_*x*_ matrix (*x* < 5/2). The filamentous Ni is ingrained in the NbO_*x*_ matrix and partially exposed to the atmosphere. Importantly, the filamentous Ni on the catalyst surface retained both the network structure and thickness even after the 550 h of gas exposure (Fig. 2e, see high-quality images in Fig. S7†). The long-term catalytic performance of the Ni_3_Nb catalyst (see Fig. 1b) is ultimately due to the stability of the nanophase-separated structure on the surface, where filamentous Ni was immobilized in the NbO_*x*_ matrix, preventing thermal agglomeration. Indeed, artificially prepared Nb_2_O_5_-supported Ni nanoparticles exhibited lower NO remediation activity and shorter lifetime (Fig. S8†) because the Ni nanoparticles are dispersed over the support surface and not embedded by the Nb_2_O_5_ matrix.

**Fig. 3:**
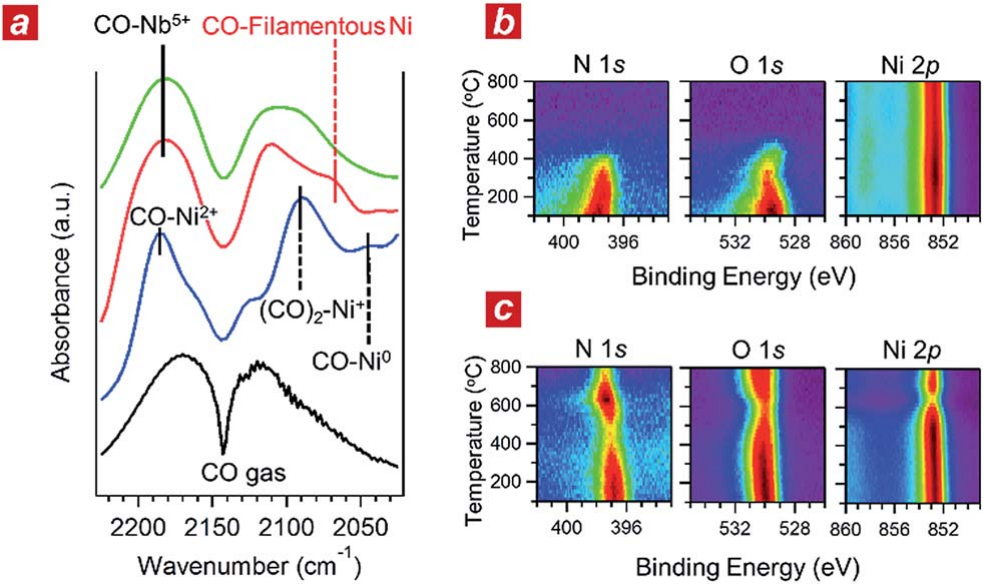
*In situ* investigations of the reaction kinetics. (a) *In situ* Fourier transformation infrared (FTIR) spectra of the Ni (blue), nanophase-separated Ni_3_Nb (red) and Nb_2_O_5_ (green) acquired in the He-balanced reactant gas at 400 °C. (b) *In situ* X-ray photoemission (XPS) spectra for the (111) surface of single-crystalline Ni that was exposed to a monolayer of NO adsorption under ultra-high vacuum (UHV). The measurement temperature ranged from 100 to 800 °C. (c) *In situ* XPS spectra for the Ni_3_Nb surface acquired under the same conditions as those in (b).

In addition, we performed *in situ* Fourier transform infrared spectroscopy (FTIR) and *in situ* XPS to elucidate the reaction kinetics behind the activity of the nanophase-separated Ni_3_Nb catalyst. As shown in Fig. 3a, when subjected to the reaction atmosphere (NO : CO : He = 1 : 1 : 98, 400 °C), the Ni catalyst accepted oxygen adatoms to form Ni^2+^O- and Ni_2_^+^O species on the surface. The FTIR peak at 2044 cm^–1^, which was assigned to the CO admolecules on the metallic Ni^0^ sites, was much less intense than the peaks at 2091 or 2183 cm^–1^ that corresponded to the CO admolecules on the Ni^+^- or Ni^2+^ sites, respectively.^23,24^

In contrast, CO molecules were adsorbed on the nanophase-separated Ni_3_Nb at metallic Ni^0^ sites, resulting in an intense IR absorption at 2071 cm^–1^. No peaks corresponding to either Ni^+^–CO or Ni^2+^–CO were observed, instead, a single peak corresponding to Nb^5+^–CO was recognized at 2183 cm^–1^.^25^ It is important to note that the Ni^0^–CO band for the nanophase-separated Ni_3_Nb catalyst had a higher wavenumber (2071 cm^–1^) than the reported Ni^0^–CO band for the pure Ni surface (2044 cm^–1^). The CO admolecules are more weakly adsorbed on the filamentous Ni than those on the pure Ni surface. These weakly adsorbed CO molecules can migrate over the surface of the filamentous Ni to efficiently scavenge oxygen adatoms.

Fig. 3b shows the results of *in situ* XPS analysis for a single-crystalline Ni(111) surface (purchased from Surface Preparation Laboratory Co.) subjected to a monolayer of NO adsorption in an ultra-high vacuum (UHV). Intense N 1s- and O 1s photoemission peaks, which correspond to the dissociative adsorption of NO, were observed at 100 °C at binding energies of 397.7 eV and 529.6 eV, respectively.^26^ Both the N 1s- and O 1s peaks became weak with increasing temperature and disappeared at around 400 °C due to the desorption of nitrogen and oxygen adatoms, respectively. The Ni 2p emission retained the peak position at 852.8 eV and became more intense as the adatoms desorbed from the surface.

The trend in the NO ad/desorption on the Ni_3_Nb surface was the NO-adsorbed Ni_3_Nb surface was located at a binding energy of 396.9 eV at 100 °C, indicating that the NO admolecules dissociated into nitrogen and oxygen adatoms. This N 1s binding energy (*i.e.*, 396.9 eV) is consistent with that for niobium nitride (NbN), which indicates that the nitrogen adatoms are not bound to Ni atoms but to Nb atoms.^27^ The N 1s emission became weaker as the temperature increased from 100 to 400 °C. However, this emission increased in intensity again at higher temperatures with a maximum at 600 °C.

This behaviour in the N 1s emission indicates that in contrast to those on the Ni surface, the nitrogen adatoms on the Ni_3_Nb surface can migrate more deeply into the bulk at approximately 400 °C than the probing depth of XPS (*i.e.*, <1 nm). The O 1s and Ni 2p emissions exhibited different behaviours than that observed for the N 1s emission. The O 1s and Ni 2p emissions were weakened at 600 °C and became intense again at higher temperatures. This trend was due to the nitrogen atoms, which migrate into the NbO_*x*_ matrix at 400 °C, being donated back to the surface of the filamentous Ni at higher temperatures, resulting in decreased Ni and O emissions.

**Fig. 4:**
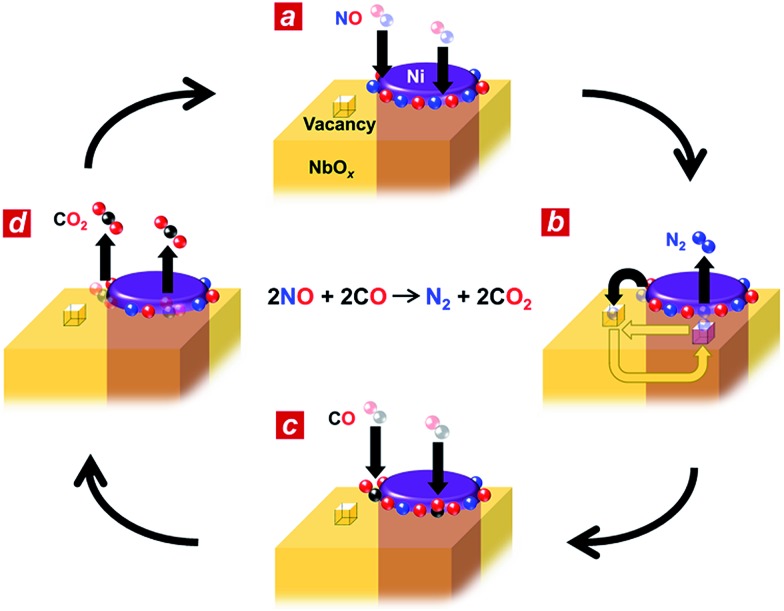
Possible reaction mechanism for the Ni_3_Nb catalyst. (a) Nanophase-separated Ni_3_Nb catalyst in the exhaust atmosphere. Both the Ni surface and metal/oxide interface perimeter are occupied by dissociative NO admolecules. (b) Oxygen vacancies on the NbO_*x*_ matrix adopt nitrogen adatoms. The nitrogen atoms are donated back to the metal/oxide perimeter to generate N_2_ molecules. (c) CO molecules are adsorbed at the free perimeter and (d) oxidized to CO_2_ by the neighbouring oxygen adatoms.

Based on the *in situ* spectroscopic results, we propose a possible reaction mechanism for the nanophase-separated Ni_3_Nb catalyst (Fig. 4). First, dissociative adsorption of NO on the surface of the filamentous Ni generates nitrogen and oxygen adatoms, which coat the exposed surface of the filamentous Ni (Fig. 4a). The nitrogen adatoms spill over to the surrounding NbO_*x*_ matrix at the metal/oxide interface, creating active Ni^0^ sites for adsorption of CO molecules. The oxygen vacancy of the NbO_*x*_ matrix, the existence of which was demonstrated by HAXPES (Fig. S3†), accommodates the nitrogen atoms due to the ability of Nb to form N–Nb bonds that are as strong as O–Nb bonds, resulting in the formation of stable oxynitrides (NbON) (Fig. 4b).^28^ The nitrogen atoms migrate through the NbO_*x*_ matrix and are donated back to the perimeter of the filamentous Ni to promote N_2_ formation. This process occurs in an analogous fashion to hydrogen spillover from metal nanoparticles to migrate through the supporting materials (*e.g.*, hydrogen atoms on Ru/[Ca_24_Al_28_O_64_]^4+^(e^–^)^4^).^29^ Then, two CO molecules are adsorbed by the free Ni^0^ sites created by N_2_ generation (Fig. 4c) and further oxidized by neighbouring oxygen adatoms to form two CO_2_ molecules (Fig. 4d). The CO_2_ formation creates four free Ni^0^ sites at the perimeter of the Ni/NbO_*x*_ interface where dissociative adsorption of two NO molecules occurs to complete the catalytic cycle.

## Conclusions

In conclusion, we have found that nanophase-separated Ni_3_Nb exhibits superior catalytic performance compared to that of Pt for NO remediation in the presence of CO. This nanophase-separated Ni_3_Nb efficiently catalyses NO remediation through the promoted absorption/back-donation of nitrogen atoms at the metal/oxide interface. It is also worth of noting that the Ni@NbO_*x*_ can promote the desired NO remediation even in the presence of oxygen (Fig. S9†). The nanophase-separated Ni_3_Nb is highly tolerant to thermal agglomeration even during long-term catalysis at elevated temperatures because the filamentous Ni phase is stably incorporated in the NbO_*x*_ matrix. The evolution of the nanophase-separated structures and catalytic functionalities may not be limited to Ni_3_Nb but could be expanded to a broad range of alloy systems containing elements with different affinities to oxidative atmospheres, such as Cu and Ni/Mn alloy materials,^30^ to produce more earth-abundant and high-performance automobile catalysts to help us meet current environmental challenges.

## Notes

T. Tanabe and T. Fujita conducted TEM/STEM characterizations. T. Tokunaga and S. Arai and Y. Yamamoto carried out *in situ* TEM characterization. S. Ueda conducted HAXPES measurements. Catalytic performance tests were done by T. Tanabe, T. Imai and H. Abe. T. Tanabe and G. V. Ramesh prepared control catalysts. *In situ* XPS was carried out by S. Nagao, H. Hirata and S. Matsumoto. T. Fujita and H. Abe equally contributed to this work through direction of whole of the research and edition of the manuscript.
