# Supplementary material for: Nanophase-separated Ni3Nb as an automobile exhaust catalyst
Source: Chem Sci. 2017 Mar 13;8(5):3374–8. doi: 10.1039/c6sc05473k (PMC5416907; doi:10.1039/c6sc05473k)
Supplement: Supplementary file 1 [file SC-008-C6SC05473K-s001.pdf]

Electronic Supplementary Material (ESI) for Chemical Science.
This journal is © The Royal Society of Chemistry 2017

Supporting Information

Title:

Nanophase-separated Ni_3Nb as Automobile Exhaust Catalysts

Toyokazu Tanabe, Tsubasa Imai, Tomoharu Tokunaga, Shigeo Arai, Yuta Yamamoto, Shigenori Ueda, Gubbala V. Ramesh, Satoshi Nagao, Hirohito Hirata, Shin-Ichi Matsumoto, Takeshi Fujita and Hideki Abe**

Contents

Experimental details 2

Figures

Figure S1: Powder X-ray diffraction (*p*XRD) profiles for different Ni intermetallics. 5

Figure S2: Hard X-ray photoemission (HAXPES) spectra for different Ni intermetallics. 6

Figure S3: HAXPES spectrum in the Nb 3*d* region for the nanophase-separated Ni_3Nb. 7

Figure S4: HAXPES spectrum in the Ni 2*p* region for the nanophase-separated Ni_3Nb. 8

Figure S5: *p*XRD profile for the Ni_3Nb material exposed to the reactant gas. 9

Figure S6: Annular-dark-field image and bright-field image of the nanophase-separated Ni_3Nb. 10

Figure S7: Cross-section TEM images of the nanophase-separated Ni_3Nb. 11

Figure S8: NO remediation activity of different catalysts. 12

Figure S9: NO_x remediation over the nanophase-separated Ni_3Nb in O_2-containing atmosphere. 13

Guide to a supporting movie 14

Experimental Details

Preparation of the samples

The samples were synthesized from elemental metals (99.9 %) in a pure Ar atmosphere (99.9999 %) using an arc torch. The prepared ingots were ground with a mortar and sieved to obtain powder samples with an average size of 50 μm. The powder of the elemental metals (99.9 %) including Pt, Ni, Nb, Nb_2O_5 was used as purchased in the catalytic tests. The samples were synthesized from elemental metals (99.9 %) in a pure Ar atmosphere (99.9999 %) using an arc torch. The prepared ingots were ground with a mortar and sieved to obtain powder samples with an average size of 50 μm. The powder of the elemental metals (99.9 %) including Pt, Ni, Nb, Nb_2O_5 was used as purchased in the catalytic tests.

Nb_2O_5-supported Ni nanoparticles were synthesized as a control catalyst (Fig. S8) by the co-precipitation method. Aliquot of 500 mg of Nb_2O_5 (Wako, 99.9 %), which was calcined at 500 °C for 5 h in air, was added to 50 ml of 0.085 M Ni chloride ($NiCl_2$, 99 % Aldrich) aqueous solution under stirring for 30 min at 80 °C. Aliquot of 5 ml of 0.8 M $NaBH_4$ (99.9 %, Aldrich) aqueous NaOH solution (0.2 g of NaOH, 99.9 % Aldrich) was added to the Ni precursor solution. After stirred for 2 h at 80 °C and collected, the final product was collected and washed several times subsequently by water and methanol. Finally, the product was dried under vacuum at room temperature overnight to obtain the desired Nb_2O_5-supported Ni nanoparticles (Ni loading = 5 wt%).

Sample characterization

Powder X-ray diffraction (*p*XRD) was performed using Cu *Kα* radiation (λ = 0.15418 nm; X'Pert Diffractometer, Panalytical) with an increment of 0.02 degrees. An obliquely finished Si crystal (non-reflection Si plate) was used as a sample holder to minimize the background. Hard X-ray photoemission spectroscopy (HAXPES) was performed using X-rays with a

photon energy of 5.95 keV at the undulator beamline BL15XU of SPring-8, Japan. The HAXPES measurements were conducted at room temperature under UHV using a hemispherical electron energy analyser (VG SCIENTA R4000). The total energy resolution was set to 220 meV. The binding energy was referenced to the Fermi edge of a Au thin film.

We used a 200 kV transmission electron microscope (TEM and/or STEM, JEM-2100F, JEOL) equipped with two aberration correctors (CEOS GmbH) for the image- and probe-forming lens systems as well as an X-ray energy-dispersive spectrometer (JED-2300T, JEOL) for compositional analyses. Both aberration correctors were optimized to achieve point-to-point resolutions of 0.13 and 0.11 nm for TEM and scanning transmission electron microscopy (STEM), respectively. A probe convergence angle of 29 mrad and a high-angle annular-dark-field (HAADF) detector with an inner angle greater than 100 mrad were used for the HAADF-STEM observation. Another TEM (JEM-2100F) was used for cross-sectional observation of the catalysts. A specially designed ion slicer was employed to prepare thin slices of the catalyst at the temperature of liquid nitrogen to minimize damage to the sample.[1] We used a JEM-1000K RS TEM (JEOL) at Nagoya University, Japan, which was equipped with a specially designed atmosphere-controllable cell, for *in situ* TEM. The point-to-point resolution of this TEM was 0.15 nm. The beam flux was measured to be 0.23 Acm^{-2} using a Faraday cup. The samples were observed in a mixture of NO and CO gas (NO:CO:Ar=1:1:98) at a constant temperature of 400 °C.

Catalytic tests

The NO remediation performance of the different catalysts was evaluated with a gas-circulation reactor and a fixed-bed flow reactor. Aliquots consisting of 50 mg and 500 mg of the powder samples were introduced into the reaction tubes (4 mm in diameter) of the gas-circulation reactor and the fixed-bed flow reactor, respectively. Aliquots consisting of a 10 kPa mixture of NO+CO gas (NO:CO=1:1) and a 100 kPa mixture of NO+CO+He gas (NO:CO:He=1:1:98 or NO:CO:O_2:He=2:4:1:93; feeding rate = 5 $cm^3\ min^{-1}$; space velocity =

30000 h^{-1}) were used for the gas-circulation and fixed-bed flow tests, respectively. The composition of the effluent gas was monitored using a gas chromatograph (Shimadzu G8-A) and a FTIR spectrometer equipped with a gas cell unit (Shimadzu Prestige 21).

Surface-chemistry analyses

The number of active Ni sites on the Ni_3Nb surface was determined by CO chemisorption with an automatized chemisorption apparatus (Autochem, Micromeritics). The TOF value was calculated by dividing the conversion rate by the number of active Ni sites. The Ni_3Nb sample was subjected to a stream of H_2 at 150 °C prior to CO chemisorption.

In situ FTIR was performed in reflectance mode using an FTIR spectrometer (FT/IR-670 plus, JASCO) equipped with an atmosphere-controllable chamber. An aliquot consisting of a 100 mg sample was placed on a carbon holder, which was introduced into the atmospheric chamber. The FTIR measurements were obtained at 400 °C in a stream of He-balanced reactant gas (NO:CO:He=1:1:98) or in an Ar atmosphere. The FTIR data acquired in the reactant gas was divided by the data in the Ar atmosphere to eliminate the background.

In situ XPS was performed on an XPS spectrometer (ULVAC PHI-1600; Pass energy = 46.95 eV, step = 0.1 eV, time/step = 50 ms) that was equipped with an ultra-high vacuum chamber and a NO gas feedthrough. The sample surfaces were cleaned under UHV by repeated Ar sputtering and heating up to 800 °C. The cleaned samples were subjected to a monolayer dose of NO gas at room temperature (gas exposure: 3.76×10^{-8} Torr for 45 s). The XPS spectra were obtained in the N 1*s*, O 1*s*, Ni 2*p* and Nb 3*d* regions at different temperatures ranging from 100 to 800 °C using Al *Ka* radiation. The temperature ramping rate was 2 °C min^{-1}.

Figures

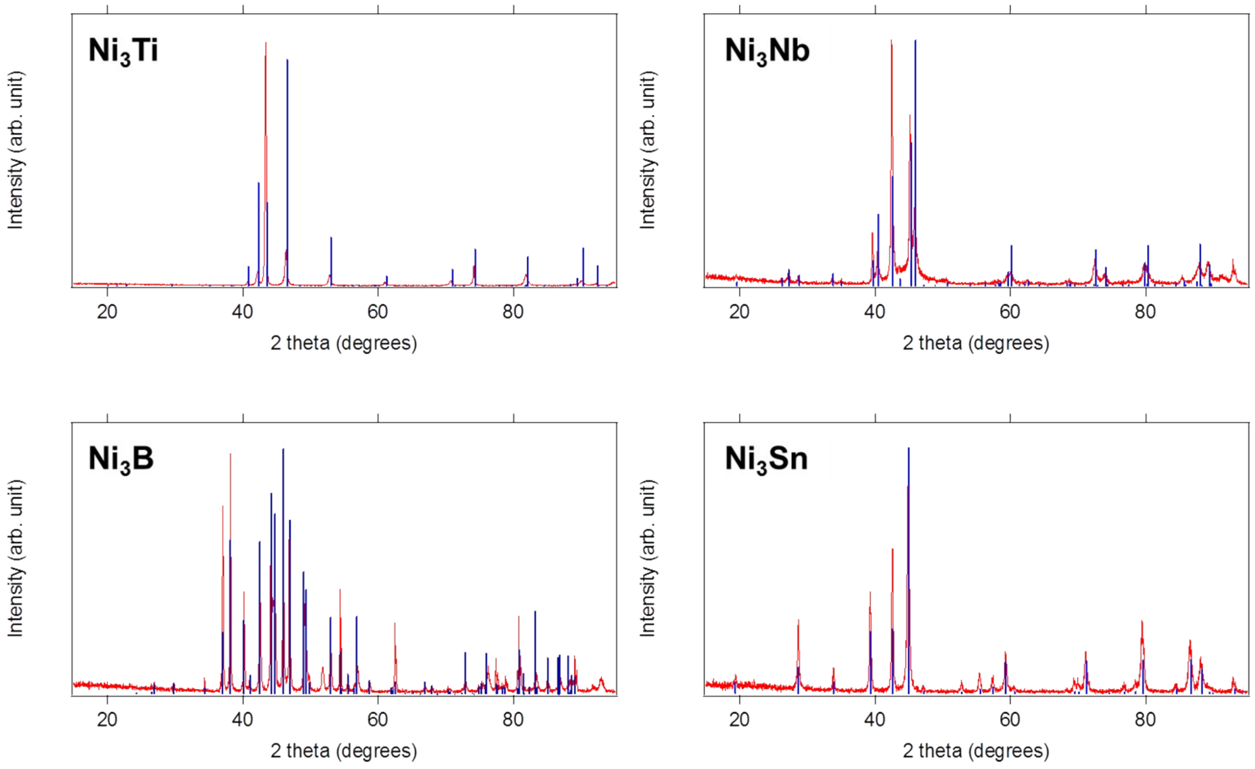

Figure S1. Powder X-ray diffraction (*p*XRD) profiles for different Ni intermetallics. *p*XRD profiles for Ni_3Ti, Ni_3Nb, Ni_3B and Ni_3Sn. Simulated *p*XRD patterns are presented as blue bars. The simulated patterns are consistent with the experimentally acquired *p*XRD profiles, indicating that the synthesized intermetallics are uniformly ordered in the desired phases.

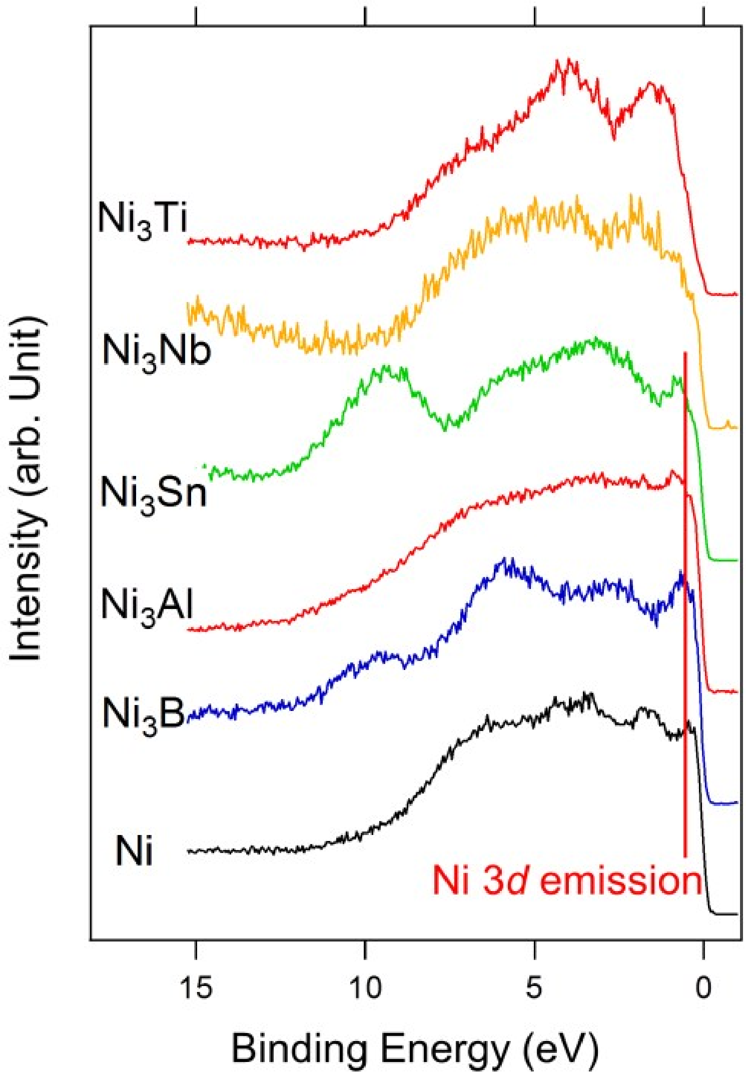

Figure S2. Hard X-ray photoemission (HAXPES) spectra for different Ni intermetallics. The HAXPES spectra for Ni, Ni_3B, Ni_3Al and Ni_3Sn show sharp Ni 3*d* emission peaks right below the Fermi level (Binding Energy = 0.0 eV). By contrast, the spectrum for neither Ni_3Ti nor Ni_3Nb shows Ni 3*d* emission peaks due to hybridization between the Ni 3*d* orbital and the Ti 3*d*- or Nb 4*d* orbitals.

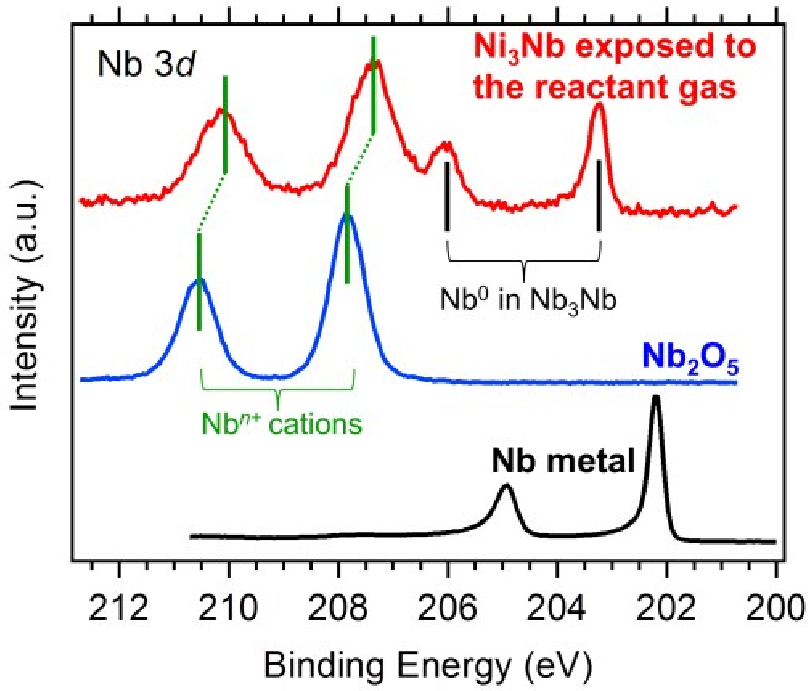

Figure S3. HAXPES spectrum in the Nb 3*d* region for the Ni_3Nb catalyst.

The Ni_3Nb catalyst was exposed to the reactant gas (NO:CO = 1:1; total pressure = 10 kPa) at 400 °C for 1 hr, forming a nanophase-separated structure on the surface. The HAXPES spectra for Nb metal and Nb_2O_5 are presented as references. The Nb 3*d* emissions from the Nb^{n+} cations in the nanophase-separated Ni_3Nb had lower binding energies than those from $Nb^{5+}{}_2O_5$, indicating that the nanophase-separated structure consists of oxygen-deficient NbO_x ($x < 5/2$).

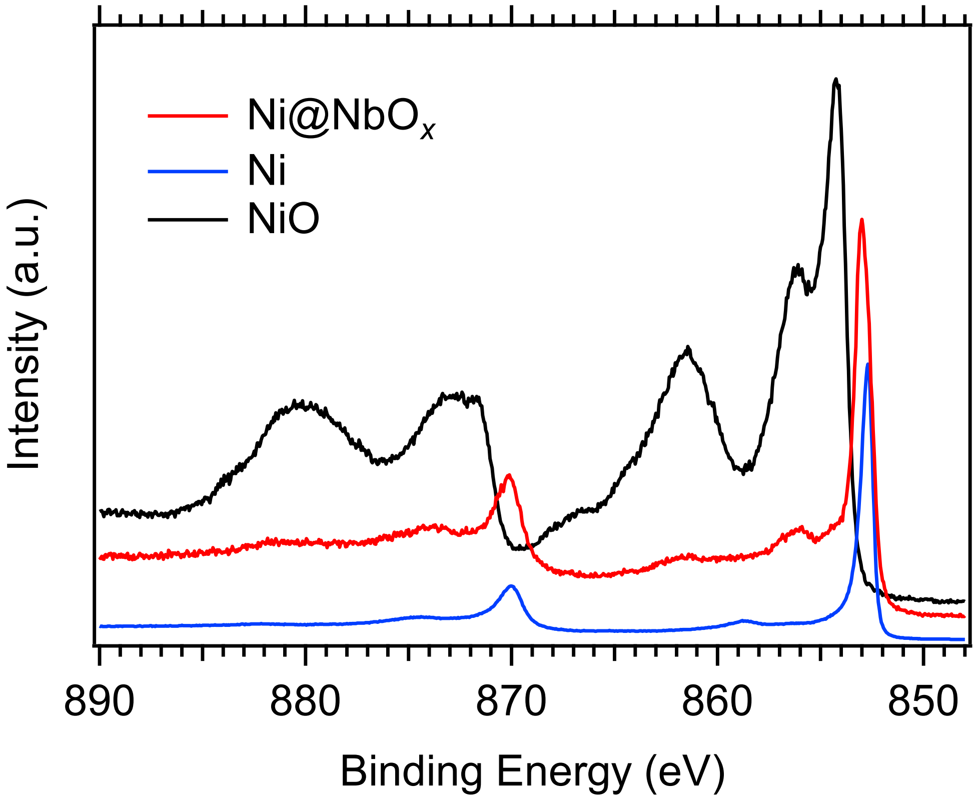

Figure S4. HAXPES spectrum in the Ni 2*p* region for the Ni_3Nb catalyst.

The HAXPES spectra for Ni metal and NiO are presented as references.

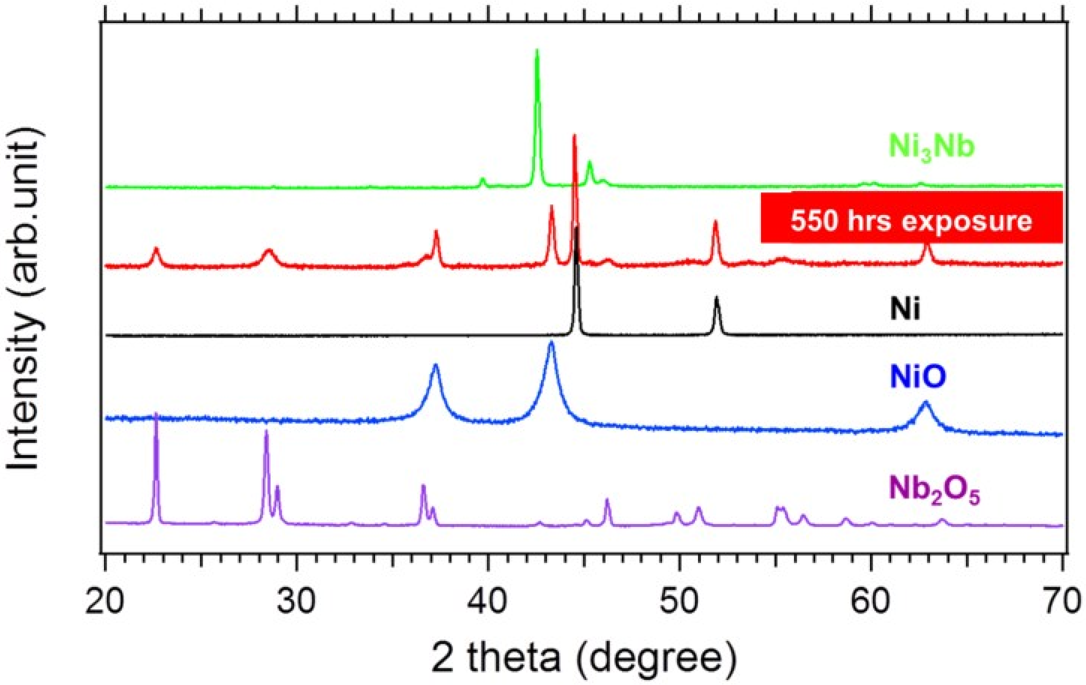

Figure S5. ***p*XRD profile for the nanophase-separated Ni_3Nb catalyst.**

The Ni_3Nb catalyst was exposed to a He-balanced reactant gas (NO:CO:He = 1:1:98) at 400 °C for 550 hrs. The *p*XRD profiles for the as-prepared Ni_3Nb, Ni, NiO and Nb_2O_5 are presented as references.

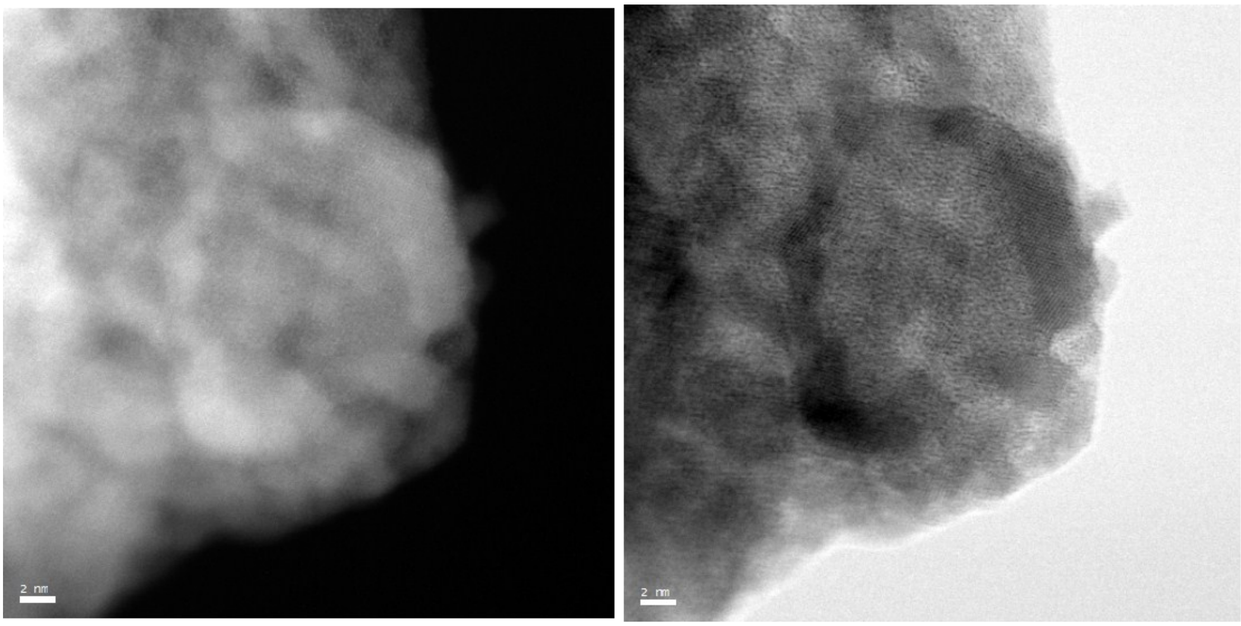

Figure S6. Annular-dark-field image (left) and bright-field image of the nanophase-separated Ni_3Nb.

The Ni_3Nb sample was exposed to the reactant gas (NO:CO = 1:1; total pressure = 10 kPa) at 400 °C for 1 hr.

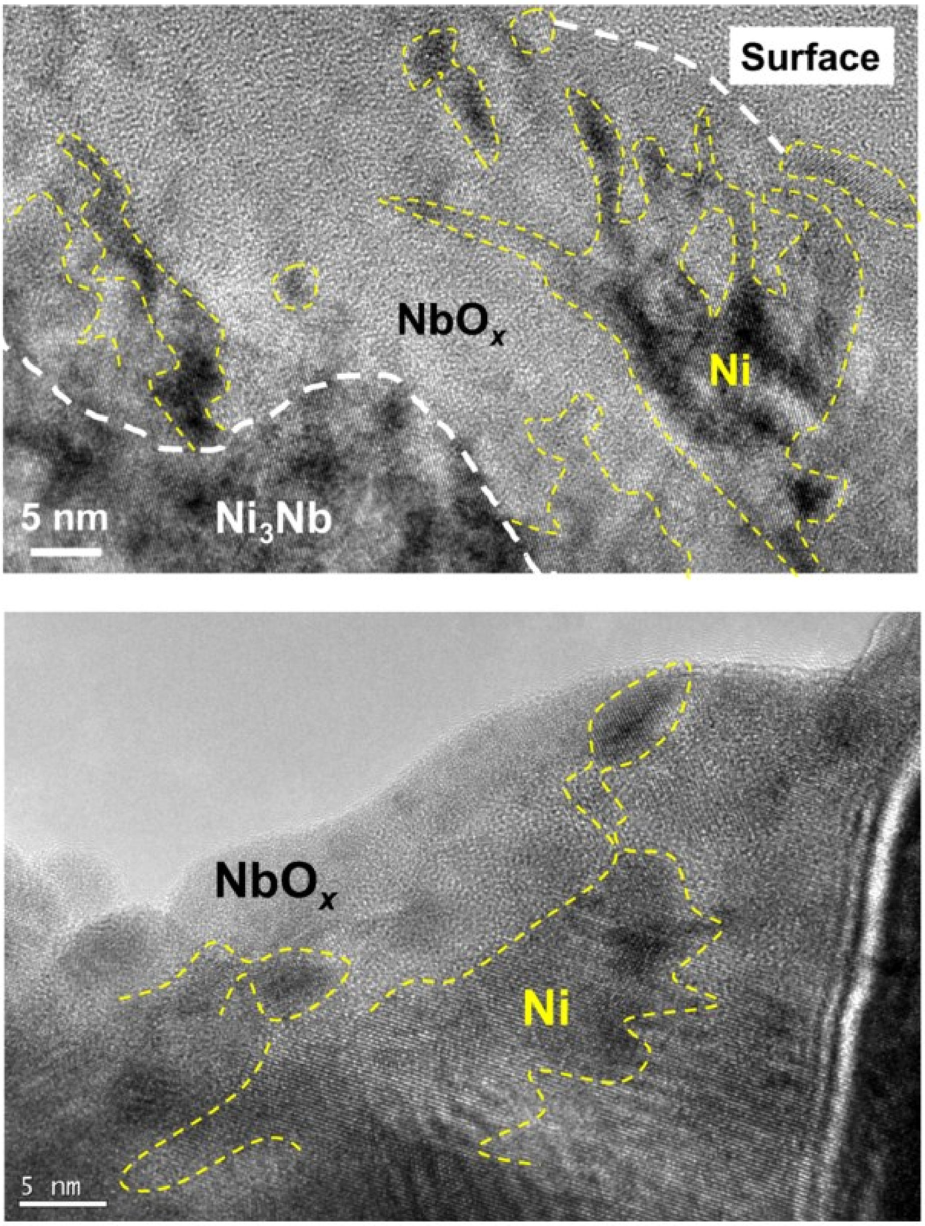

Figure S7. Cross-section TEM images of the nanophase-separated Ni_3Nb.

The images correspond to the Ni_3Nb catalysts after 1 hr of exposure (top) and after 550 hrs of exposure (bottom) to the He-balanced reactant gas (NO:CO:He = 1:1:98; 400 °C; see the text).

[1] T. Tanabe, *Microscopy* **2011**, *60*, 35.

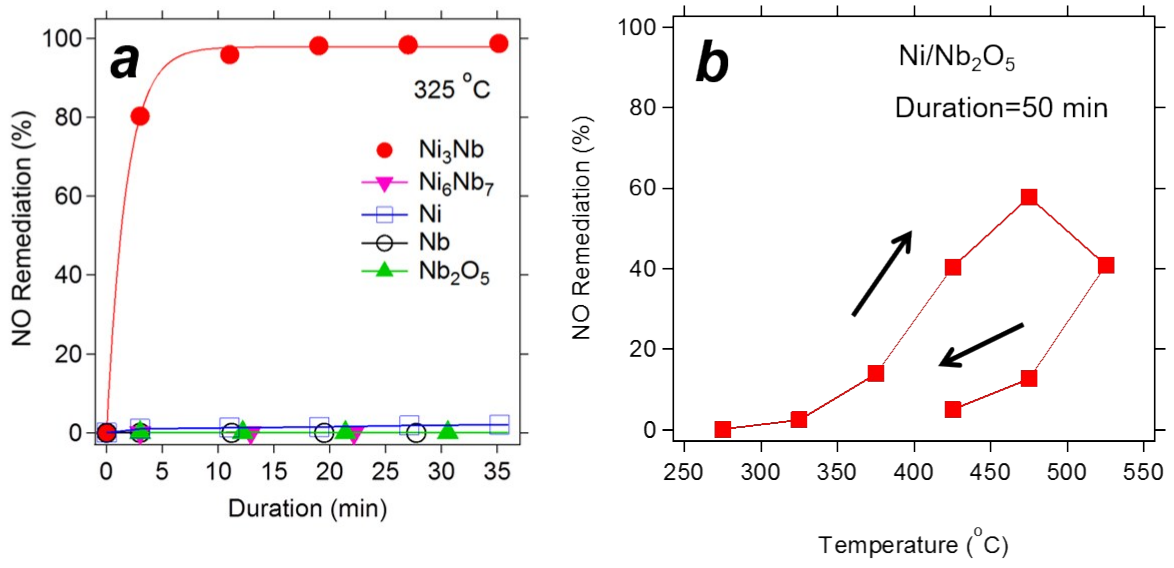

Figure S8. NO remediation activity of different catalysts. Catalytic tests were performed at 325 °C in a simulated exhaust gas (NO:CO = 1:1; total pressure = 10 kPa) with a circulation-gas reactor that was used for Fig.1a in the main text. **a,** Catalytic activities of Ni, Ni_6Nb_7, Nb, Nb_2O_5 and Ni_3Nb. The Ni, Nb, Nb_2O_5 catalysts were used as purchased from Aldrich. **b,** Catalytic activity of Nb_2O_5-supported Ni nanoparticles at different temperatures. Each of the NO-remediation activty data was evaluated at 50 min. after the catalyst was exposed to the simulated exhaust gas. Note that the NO-remediation activity evaluated at 425 °C in decreasing temperature was lower than that in increasing temperature, indicating that the catalyst was degraded during the catalytic reactions up to 525 °C.

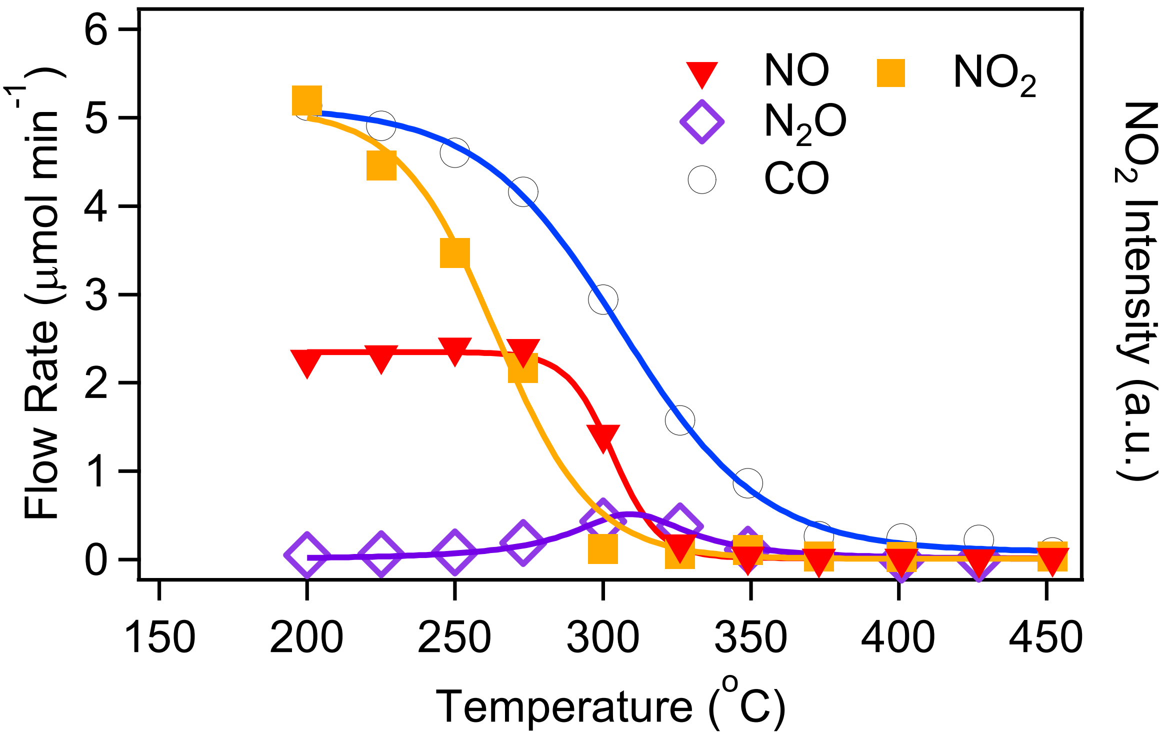

Figure S9. NO_x remediation over the nanophase-separated Ni_3Nb in O_2-containing atmosphere (O_2:CO:NO:He=1:4:2:93 vol.%). The flow rates for the NO-, N_2O- and CO gases are plotted as functions of temperature (left axis). The right axis corresponds to the concentration of NO_2 in the effulent gas.

Guide to a Supporting Movie:

Title: Evolution of the nanophase-separated structure

Caption: A video stream showing the evolution of the nanophase-separated structure on the surface of the Ni_3Nb intermetallic phase that was exposed to an Ar-balanced reactant gas (NO:CO:Ar = 1:1:98) at 400 °C. The video frequency was 290 fps. The width of the video frame corresponds to 400 nm. The starting time of the video corresponds to 1.5 min after exposure to the reactant gas. See a series of capture images presented in the main text (Fig. 2*a*).
